# A Stereo Synchronization Method for Consumer-Grade Video Cameras to Measure Multi-Target 3D Displacement Using Image Processing in Shake Table Experiments

**DOI:** 10.3390/s25175535

**Published:** 2025-09-05

**Authors:** Mearge Kahsay Seyfu, Yuan-Sen Yang

**Affiliations:** Department of Civil Engineering, National Taipei University of Technology, No. 1, Sec. 3, Zhongxiao E. Rd., Daan Dist., Taipei 10608, Taiwan; t112429402@ntut.edu.tw

**Keywords:** image processing, synchronization, 3D displacement, accuracy, shake table, software

## Abstract

The use of consumer-grade cameras for stereo vision provides a cost-effective, non-contact method for measuring three-dimensional displacement in civil engineering experiments. However, obtaining accurate 3D coordinates requires accurate temporal alignment of several unsynchronized cameras, which is often lacking in consumer-grade devices. Current synchronization software methods usually only achieve precision at the frame level. As a result, they fall short for high-frequency shake table experiments, where even minor timing differences can cause significant triangulation errors. To address this issue, we propose a novel image-based synchronization method and a graphical user interface (GUI)-based software for acquiring stereo videos during shake table testing. The proposed method estimates the time lag between unsynchronized videos by minimizing reprojection errors. Then, the estimate is refined to sub-frame accuracy using polynomial interpolation. This method was validated using a high-precision motion capture system (Mocap) as a benchmark through large- and small-scale experiments. The proposed method reduces the RMSE of triangulation by up to 78.79% and achieves maximum displacement errors of less than 1 mm for small-scale experiments. The proposed approach reduces the RMSE of displacement measurements by 94.21% and 62.86% for small- and large-scale experiments, respectively. The results demonstrate the effectiveness of the proposed method for precise 3D displacement measurement with low-cost equipment. This method offers a practical alternative to expensive vision-based measurement systems commonly used in structural dynamics research.

## 1. Introduction

The precise measurement of structural response during dynamic loading events represents a fundamental challenge in earthquake engineering, where precise quantification of displacement, deformation, and rotational behavior is essential for understanding structural performance and developing robust design methodologies [1]. Although traditional contact-based measurement systems offer high accuracy [2], they impose significant limitations in terms of practical applicability and cost in dynamic testing environments [3,4]. The mounting complexity of conventional sensors necessitates substantial preparation time and expertise [5]; their limited dynamic range may fail to capture the full spectrum of structural response during extreme loading events [6]. Furthermore, these systems are vulnerable to damage during high-intensity dynamic tests, such as shake table experiments, where sensors are possibly subjected to severe structural deformations and accelerations. The emergence of vision-based measurement techniques, enabled by advances in computational capacity and camera technology, has introduced transformative possibilities for non-contact structural monitoring and experimental characterization [7,8]. These methodologies leverage computer vision and image processing algorithms to capture three-dimensional displacement, deformation, vibration, and strain fields of structures and components under dynamic loading conditions. Recent developments in intelligent structural health monitoring have further demonstrated the potential of integrating computer vision with edge computing technologies to enable real-time monitoring capabilities [9,10]. Unlike traditional measurement approaches, vision-based systems eliminate physical contact with test specimens, thereby avoiding sensor damage while enabling comprehensive spatial measurement capabilities that extend beyond the unidirectional constraints of conventional devices. The advantages of vision-based measurement become particularly pronounced when investigating freely moving structural and non-structural components, such as suspended ceiling systems that may experience large displacements, overturning, bending, collapse, and complex three-dimensional rotations during seismic excitation [11]. Such scenarios present insurmountable challenges for traditional measurement systems, highlighting the critical need for advanced non-contact measurement capabilities in contemporary structural engineering research.

Vision-based measurement has promising theoretical advantages [12], but its real-world application in dynamic structural testing is still catching up. Most studies have not explored its potential in shake table experiments, leaving this area relatively underexplored. Ngeljaratan and Moustafa [13] observed a 5–7% overestimation of peak response values using image-based measurement compared to linear variable differential transformer (LVDT) measurements in a dynamic test of a four-story steel frame building. This finding indicates an inherent challenge in achieving measurement accuracy levels comparable to established contact-based techniques. Wani et al. [14] employed template matching algorithms in the analysis of the response of a five-story damped steel-frame building under shake table ground acceleration, with maximum displacement measurement deviations of 6.142 mm from the reference. While these results do substantiate feasibility, they indicate that improvements in algorithmic approaches and measurement processes are needed. On the contrary, Sieffert et al. [15] reported more positive results in their investigation of a single-story timber-frame building filled with earth and stone under seismic load, employing Digital Image Correlation (DIC) software called “Tracker” to achieve displacement measurement differences of less than 5% compared to results obtained from LVDT measurements. Such different results indicate the radical influence that test conditions, target properties, and measuring parameters exert on system performance.

Stereo vision-based 3D measurement technologies typically comprise four essential processes: camera calibration, target tracking, temporal synchronization, and stereo triangulation. To achieve precise geometric reconstruction, camera calibration involves adjusting for lens distortion and measuring intrinsic and extrinsic camera parameters. Target tracking involves the temporal detection and localization of measurement points within image sequences, while temporal synchronization guarantees synchronized data acquisition from two or more cameras. Stereo triangulation computes 3D coordinates through the intersection of corresponding image points. While these processes constitute the foundation of stereo vision, recent advances in computer vision showed that monocular approaches can also recover 3D measurements [2,6,16], gaining attention for their simplified hardware requirements and cost-effectiveness. However, monocular methods remain limited in out-of-plane displacement accuracy due to restricted depth perception. In contrast, stereo vision offers better geometric accuracy via triangulation but requires a more complex setup.

Among the four stereo processes, temporal synchronization remains particularly challenging for consumer-grade equipment. While recent computer vision advances have achieved subpixel spatial accuracy for single-camera vibration measurement [17,18], these approaches address spatial precision within individual video streams rather than temporal synchronization between multiple cameras. These methods operate on single-camera data and do not address the sub-frame temporal alignment required for accurate stereo triangulation in multi-target displacement measurement. The current study addresses this distinct problem: achieving sub-frame temporal synchronization between consumer-grade cameras for multi-target 3D displacement measurement in structural testing applications.

The measurement accuracy of a dynamic vision-based measurement system depends on a complex interaction of numerous factors that must be fine-tuned with caution for specific experimental conditions. The critical parameters are the selection of the tracking algorithm [19], geometry and material properties of markers [20], resolution and optical characteristics of cameras [21], sampling rate of video, illumination, lens quality, calibration procedures [22], target speed [23], surface characteristics of the specimen, environmental factors [24,25], camera stability [26], working distance, and angle of view [27,28]. Recent studies have also emphasized the need for external multimodal sensor calibration and fusion techniques to enhance the precision and reliability of measurements in advanced experimental configurations [29]. The coupled nature of these variables presents a multidimensional optimization issue that necessitates rigorous experimental design and validation.

Multi-camera synchronization is the most critical technical challenge limiting the widespread adoption of vision-based measurement in dynamic structural testing. Precise camera synchronization is crucial for accurate 3D position determination, as even minor timing errors can significantly impact stereo triangulation [30,31]. Synchronization is crucial for reliable multi-camera multi-object tracking [32], especially when measuring rapid structural responses or complex motions. The measurement of torsional response is a particularly demanding application where precise synchronization is indispensable. Vision-based systems offer unique advantages for torsional measurement due to their ability to simultaneously survey multiple points and measure complete three-dimensional motion fields. However, synchronization errors lead to phase mismatches between the rotational data and other system parameters, which magnify or reduce the observed torsional effects, resulting in incorrect structural behavior interpretation and erroneous conclusions regarding structural stability and response characteristics under introduced loading.

Existing synchronization techniques have various efficiency levels, each with inherent limitations that restrict their application in stringent experimental environments. Manual synchronization procedures, such as the time stamp correction techniques applied by Lavezzi et al. [33], are likely to produce noisy displacement values due to the inherent inaccuracy of temporal coordination. Hardware synchronization systems [27,34] are capable of higher accuracy but must be fitted with expensive data acquisition systems that limit practical field application and contribute to overall system cost and complexity. Other approaches include using optical synchronization markers, such as synchronized light sources visible to all cameras [35]. However, these techniques do not typically provide the sub-frame-level accuracy required for high-frequency dynamic measurements. Software synchronization methods offer more universally available solutions with different performance levels. Cross-correlation methods [22] work well for synchronizing camera timing differences but are limited to the frame resolution integer, which might be too low for high-speed dynamic applications. More sophisticated methods, such as wavelet transform methods [36] and multistage synchronization protocols [37], are superior. The second approach achieves synchronization accuracy of 250 microseconds through network time protocol (NTP) versions, followed by stream phase synchronization; however, it requires specialized hardware capabilities that may not be supported by all consumer cameras. Recent advances in computer vision and multi-camera systems have underscored the importance of high-fidelity synchronization in diverse real-world applications. To derive temporal correlations between cameras, sub-frame-level synchronizations have been established using time-stamped video methods with specific markers and uniformly translating targets [38]. These developments signify the increasing recognition that synchronization accuracy has direct implications for the accuracy of vision-based measurement systems in various fields.

The imperative requirement of high-precision synchronization in the measurement of structural dynamics and the limitations of available methods constitute a key research gap that restricts the broader application of vision-based measurement approaches to earthquake engineering applications. Current synchronization techniques are inaccessible to the broader research community because they either lack the accuracy needed for dynamic structural measurements, necessitate costly specialized hardware, or rely on device capabilities that are not consistently present in consumer-level cameras. This study addresses these fundamental limitations by developing a novel synchronization methodology with sub-frame accuracy specifically designed for structural engineering applications. This study presents a comprehensive solution that integrates precise temporal alignment with a graphical user interface-based software system for measuring three-dimensional displacements of multiple targets in shake table experiments using readily available consumer-level cameras. The strategy described here aims to bridge the gap between the measurement accuracy requirements and practical constraints to facilitate the broader application of vision-based measurement methodologies in structural dynamics research. A thorough and reliable experimental validation procedure is employed to assess the accuracy and dependability of the proposed synchronization method and related image-based measurement capabilities. This study represents a significant step toward making sophisticated vision-based measurements accessible to broader structural engineering applications, from laboratory-based shake table testing to field-based structural health monitoring and post-earthquake damage reconnaissance.

## 2. Materials and Methods

This section describes the methodological workflow and experimental program used in this study. This study encompassed two distinct experimental phases. The first phase involved a physical experiment consisting of video recordings of the experiments conducted on moving targets. The second phase comprised a non-physical experiment centered on image analysis using the developed software.

### 2.1. Experimental Program

This study employed four distinct experimental setups to evaluate the accuracy of the proposed synchronization method and the developed application software in determining the 3D coordinates of multiple moving targets under various conditions. The types of experimental setups used are listed as follows.

**Figure 1:**
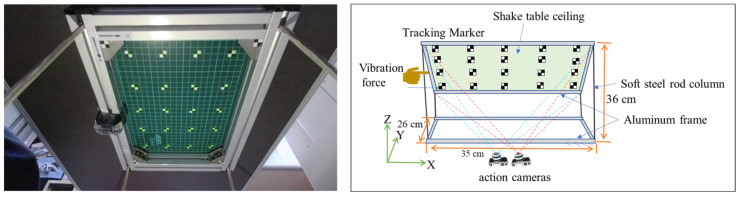
Experimental setup of the small-scale frame structure; photo (**left**) and schematic illustration (**right**).

**Figure 2:**
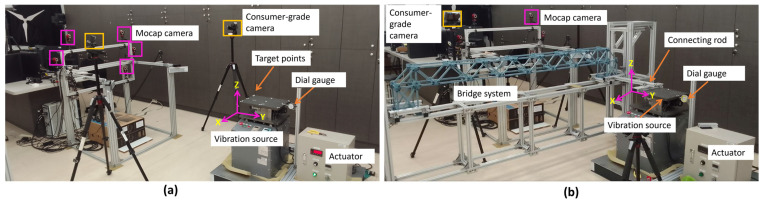
Experimental setup: (**a**) shake table only; (**b**) truss bridge connected to a shake table.

**Figure 3:**
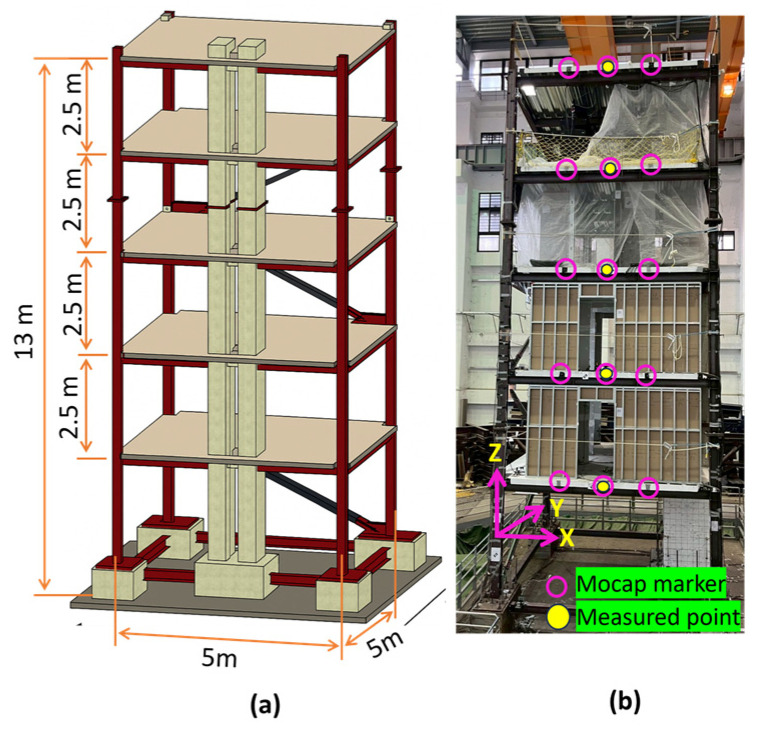
Full-scale experimental specimen: (**a**) schematic illustration; (**b**) location of measured points.

**(a)**  A small-scale frame structure (Figure 1) with dimensions 26 cm wide, 35 cm long, and 36 cm high, fixed at its base, was used to test the proposed stereo synchronization method’s accuracy. A grid of 20 tracking markers was fixed and arranged in a 4-by-5 configuration on the ceiling of the specimen. Figure 1 shows an image of the structure ceiling with tracking markers on its surface. An excitation force was applied by pushing the structure’s top edge in the horizontal direction. It vibrated freely until it stopped, and a video was recorded using two consumer-grade action cameras (SJCAM) situated beneath the structure’s ceiling with a recording video quality of 4K and a sampling rate of 60 frames per second (fps).
**(b)**  A shake table with a fixed harmonic displacement amplitude of 3.00 mm was used along with an industrial motion capture system (Mocap) [39] and a dial gauge to validate the displacement measurement accuracy of the image-based measurement system. Previous studies have investigated the accuracy of Mocap using different approaches, and they have proved that its measurement error is less than a millimeter [40,41]. The Mocap system used for validation has six Optiflex 13 cameras (NaturalPoint, Inc., Corvallis, OR, USA) positioned 2 m from the targets. Two SJCAM action cameras were used to record the experimental videos with a video quality of 4K and a frame rate of 60 fps. Figure 2a shows a schematic of the experimental setup used.
**(c)**  A truss bridge was constructed using a plastic frame and connected to a shake table via an aluminum bar, as shown in Figure 2b. To verify the accuracy of the displacement measurements obtained from the proposed method, we utilized a Mocap system. This experiment differs from the others because the tracking points were located on a secondary object (the bridge), and the specimen was not rigid. This secondary object had a unique vibration pattern, distinct from the primary vibration source (shake table), which exhibited harmonic motion at various frequencies. This helped us to validate the capability of the proposed method in capturing higher-order vibration on the bridge.
**(d)**  The proposed synchronization strategy was further validated through shake table tests conducted on a five-story full-scale test structure (see Figure 3). The El Centro earthquake ground acceleration was used as the excitation force in the experiment for both the *X* and *Y* directions. The dimensions of the structure were 5 m by 5 m, with a total height of 13 m. This experimental work took place at the National Center for Research on Earthquake Engineering in Tainan, Taiwan. Cameras were positioned approximately 12 m from the specimen, and displacement was measured on all floors. To record the experimental videos, two SJCAM action cameras with 4K video quality and a frame rate of 60 fps were used. The points at which displacement was measured on each floor are indicated in Figure 3b.

### 2.2. Accuracy Metrics

To rigorously assess the accuracy of our vision-based displacement measurement method, we compared its outputs, *Y_i_*, against Mocap *X_i_* over *N* time steps using three complementary error types. Note that *N* is the number of time steps after synchronization.

1. Maximum absolute error (*MAE*) quantifies the worst-case deviation, given by Equation (1).

(1) $${MAE=Max}_{i=1,\ldots ,N}=\left|Y_{i\;\;}-X_{i}\right|$$

2. Mean error (*ME*) measures the average magnitude of errors, given by Equation (2).

(2) $$ME=\frac{1}{N}\sum_{i=1}^{N}\left|Y_{i}-X_{i}\right|$$

3. Root mean square error (*RMSE*) emphasizes larger errors by squaring residuals before averaging. *RMSE* is particularly sensitive to outliers and provides error magnitude in the same units as the measurement. The *RMSE* equation is given by Equation (3).

(3) $$RMSE=\sqrt{\frac{1}{N}\sum_{i=1}^{N}{\left(Y_{i}-X_{i}\right)}^{2}}$$

To compare the measurement results obtained from Mocap and the proposed method, we employed a Python-based signal synchronization algorithm to align the displacement signals. This algorithm leverages cross-correlation techniques to achieve precise synchronization.

### 2.3. Image-Based Measurement Mathematical Models and Procedures

**Figure 4:**
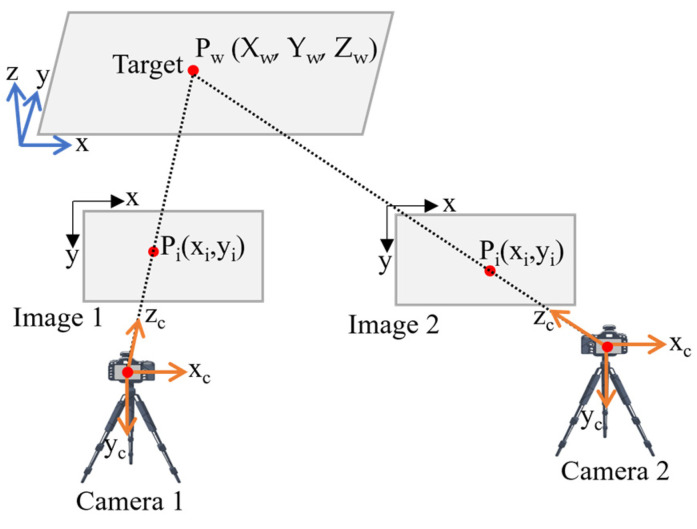
Illustration of stereo camera setup.

This study employs a stereo vision system to capture the 3D coordinates of target points in images [42], generating time series data for each target across the measurement specimen. The time history of these targets enables the estimation of displacement and vibration modes. Deformation modes, including rigid body displacement, torsion, shear, bending, and crack patterns, can be derived from the 3D coordinates of multiple points over time [22]. In a stereo vision system, transferring a point from three-dimensional space to two-dimensional images and camera coordinates necessitates geometric transformations [26]. To perform these transformations, three coordinate systems must be established as world coordinates *P_w_* = (*X_w_*, *Y_w_*, *Z_w_*), camera coordinates *P_c_* = (*x_c_*, *y_c_*, *z_c_*), and image coordinates *P_i_* = (*x_i_*, *y_i_*), as shown in Figure 4.

The coordinate transformation from the image coordinate to the world coordinate can be expressed in terms of a 4 × 4 matrix, as indicated in Equation (4). This matrix is used to account for rotation, translation, affine, and perspective transformations [43]. The 3 × 3 matrix located in the upper left corner of the 4 × 4 matrix is the rotational matrix, and the vector T = (*T_x_*, *T_y_, T_z_*)^T^ represents the translation of the world coordinate (*P_w_*) to the origin of the camera coordinate system (*x_c_, y_c_, z_c_*). Mathematically, the relation between the camera coordinate and the world coordinate can be defined using Equation (4) [43,44].

(4) $$\left[\begin{array}{c} x_{c}\\ y_{c}\\ z_{c}\\ 1 \end{array}\right]=\left[\begin{array}{cccc} R_{xx} & R_{yx} & R_{zx} & T_{x}\\ R_{xy} & R_{yy} & R_{zy} & T_{y}\\ R_{xz} & R_{Yz} & R_{zz} & T_{z}\\ 0 & 0 & 0 & 1 \end{array}\right]\left[\begin{array}{c} X_{w}\\ Y_{w}\\ Z_{w}\\ 1 \end{array}\right]$$

The geometric coordinate transformation from the camera coordinate *P_c_* = (*x_c_, y_c_, z_c_*) to the image coordinate *P_i_* = *(x_i_*, *y_i_*) can be represented using Equation (5). In this type of transformation, the computation is easy when the dimension of the coordinates in the projective space is equivalent to that of the projected space. Therefore, the coordinates on the camera screen will be represented by *P_c_* = (*x_c_*, *y_c_*, *z_c_*). The proportion of values for all points originating in a three-dimensional space, when projected onto a two-dimensional screen, remains consistent and equivalent [45]. If we divide the camera coordinates (*x_c_, y_c_, z_c_*) by a unit length *z_c_* = 1, we obtain the normalized coordinates (*x_n_, y_n_,* 1)^T^ [22]. Therefore, the image coordinates after the effect of distortion are accounted for as given by Equation (5).

(5) $$\left[\begin{array}{c} x_{i}\\ y_{i}\\ 1 \end{array}\right]=\left[\begin{array}{ccc} f_{x} & 0 & c_{x}\\ 0 & f_{y} & c_{y}\\ 0 & 0 & 1 \end{array}\right]\left[\begin{array}{c} x_{d}\\ y_{d}\\ 1 \end{array}\right]$$

where *f_x_*, *f_y_*, *c_x_*, and *c_y_* are the intrinsic camera parameters that define the characteristics of the camera, the 3 × 3 matrix containing these values is known as the camera matrix, and (*x_d_*, *y_d_*, 1)^T^ is a matrix that accounts for the effect of lens distortion on normalized coordinates. The relationship between the distorted coordinates and normalized coordinates can be defined using Equation (6) [22,45].

(6) $$\left[\begin{array}{c} x_{d}\\ y_{d} \end{array}\right]=\left[\begin{array}{cc} k+2p_{1}y_{n}+3p_{2}x_{n} & p_{2}x_{n}\\ p_{1}x_{n} & k+2p_{2}x_{n}+3p_{1}y_{n} \end{array}\right]\left[\begin{array}{c} x_{n}\\ y_{n} \end{array}\right]$$

where

(7) $$k=\frac{1+k_{1}({x_{n}}^{2}+{y_{n}}^{2})+k_{2}{\left({x_{n}}^{2}+{y_{n}}^{2}\right)}^{2}+k_{3}{\left({x_{n}}^{2}+{y_{n}}^{2}\right)}^{3}}{1+k_{4}({x_{n}}^{2}+{y_{n}}^{2})+k_{4}{\left({x_{n}}^{2}+{y_{n}}^{2}\right)}^{2}+k_{6}{\left({x_{n}}^{2}+{y_{n}}^{2}\right)}^{3}}$$

where *k*_1_, *k*_2_, *k*_3_, *k*_4_, *k*_5_, and *k*_6_ are radial distortions, and *p*_1_, and *p*_2_ are tangential distortion parameters.

The image-based measurement was conducted by employing the following four steps: (1) camera calibration, (2) tracking, (3) synchronization, and (4) stereo triangulation.

#### 2.3.1. Camera Calibration

Images captured by a camera often exhibit distortion due to the lens’s perspective features. Camera calibration corrects distortion and other parameters to obtain a rectified image. There are two types of camera parameters to consider: intrinsic and extrinsic parameters. Intrinsic parameters define how the camera captures images, whereas extrinsic parameters define the camera’s location within the 3D environment. Chessboards are most commonly used to calibrate a camera [46,47,48] because calibration algorithms can easily identify corners. However, chessboards are not reliable for large-area experiments because large, rigid calibration chessboards are not practical. Different types of markers with known world coordinates, selected from a single image frame of a video, were used as input for our calibration software, which was developed using the OpenCV computer vision library. Templates belonging to the respective targets were selected using the calibration algorithm to obtain the image coordinates. Each camera was calibrated separately. The intrinsic and extrinsic camera parameters were calculated using our program based on the relations defined in Equations (4)–(7). These equations are based on the formulations presented in [22,45].

#### 2.3.2. Tracking

**Figure 5:**
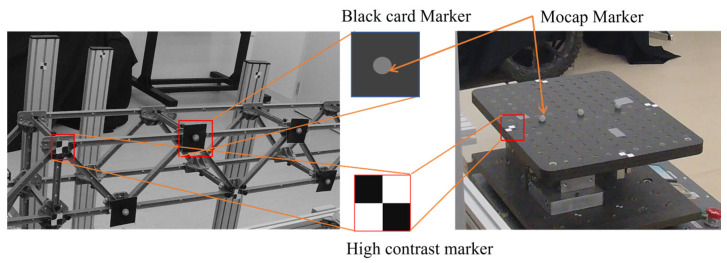
Types of markers used in this study.

Target tracking is the process of detecting and following a specific target’s movement within a sequence of images in computer vision-based measurement [49]. Having tracking points within a small area, commonly referred to as a template, is crucial in identifying the target using tracking algorithms. This template should exhibit visual characteristics that differ from those of the parent image’s surrounding elements. Templates can be created by applying paint, attaching markers, or using natural features of the measurement area, such as holes, bolts, or joints. Several types of templates were employed for the tracking task, including black-and-white high-contrast markers, holes, backgrounds with distinguishable patterns, and stickers. Figure 5 depicts some of the markers used in this study. We employed an enhanced correlation coefficient (ECC) tracking method to track the targets. ECC is a computer vision approach that tracks objects or features across a sequence of images. The ECC algorithm aligns images by optimizing zero-mean normalized cross-correlation [4], which enhances its robustness against global variations in brightness and contrast, such as differences in exposure. However, the algorithm’s accuracy diminishes in the presence of significant non-uniform illumination, including shadows or localized lighting variations. In this study, small-scale experiments were conducted under normal lighting, while full-scale experiments were conducted in a noisy lighting environment.

#### 2.3.3. Camera Synchronization

Camera synchronization is crucial for obtaining accurate three-dimensional coordinates of target objects in multi-camera vision systems. Even small timing differences between camera captures can cause major errors in triangulated 3D coordinates, which impacts measurement accuracy. The experimental cameras used in this study do not support trigger synchronization; therefore, software-based synchronization of video streams is necessary for reliable vision-based measurements. In this study, a synchronization approach was implemented to determine the optimal time lag between camera feeds by minimizing triangulation reprojection errors. The proposed algorithm identifies the temporal offset that produces the minimum mean squared error (MSE) of the triangulation projection errors across all detected points. To support this idea, Equations (8)–(11) are proposed by this study. The synchronization process operates in two phases. Initially, the algorithm searches for the coarse time lag that minimizes the overall reprojection error. Subsequently, it refines this estimate to achieve sub-frame precision using polynomial interpolation techniques. While similar methodologies have been proposed in previous research [50], this approach differs significantly in its time lag compensation implementation. Rather than interpolating between unsynchronized 3D coordinate points, the proposed method performs interpolation directly on image points. This modification enhances triangulation accuracy by maintaining the geometric relationships inherent in the imaging process. The assumption is that the triangulation projection error (*e*) is a function of time lag (*t_lag_*), expressed by a quadratic equation. If (*e*_1_, *e*_2_, *e*_3_) are the triangulation MSEs of all points at a time lag of (*t*_1_, *t*_2_, *t*_3_), respectively, we can find the optimum time lag (*t_opt_*) as shown in Equation (9) by derivation of Equation (8). Equation (8) can be expressed in matrix form as given in Equation (10).

(8) $$e=at^{2\;}+bt+c$$

(9) $$t_{opt}=\frac{de}{dt}[at^{2\;}+bt+c]=-b/2a$$

**Figure 6:**
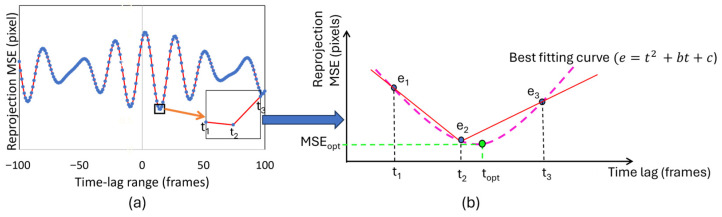
Schematic illustration of time lag determination: (**a**) frame level; (**b**) optimized (sub-frame level).

The coefficients (a) and (b) can be obtained by solving Equation (9).

(10) $$\left[\begin{array}{c} e_{1}\\ e_{2}\\ e_{3} \end{array}\right]=\left[\begin{array}{ccc} t_{1}^{2} & t_{1} & 1\\ t_{2}^{2} & t_{2} & 1\\ t_{3}^{2} & t_{3} & 1 \end{array}\right]\left[\begin{array}{c} a\\ b\\ c \end{array}\right]$$

where *t*_1_ *= t*_2_ − 1 and *t*_3_ *= t*_2_ *+* 1, with *t*_2_ representing the optimal frame-level time lag that exhibits the minimum triangulation projection error across the entire temporal range analyzed. This configuration creates a symmetric sampling window centered on the coarse optimum (*t*_2_), which provides the necessary data points for quadratic interpolation to find the best-fitting curve, as illustrated in Figure 6.

The quadratic assumption in Equation (9) follows from the mathematical structure of reprojection error within small temporal neighborhoods. For smooth trajectories, marker positions vary linearly with a temporal offset within the ±1 frame window. Since reprojection error is the square of the Euclidean distance between observed and predicted coordinates, these linear position deviations produce quadratic error scaling. This relationship is invariant across marker sizes, motion frequencies, and trajectory patterns because it derives from squaring a linear function, requiring only local motion linearity over single-frame intervals. Based on this theoretical foundation, we consistently used second-order polynomial interpolation for sub-frame refinement. The algorithm automatically selects the interpolation window, utilizing a ±1 frame window centered on the integer-frame minimum obtained from coarse synchronization. This 3-point window provides the minimum sampling for quadratic fitting while maintaining local validity around each point’s optimal lag. This ensures a consistent application across all tracking points without the need for manual parameter tuning.

**Figure 7:**
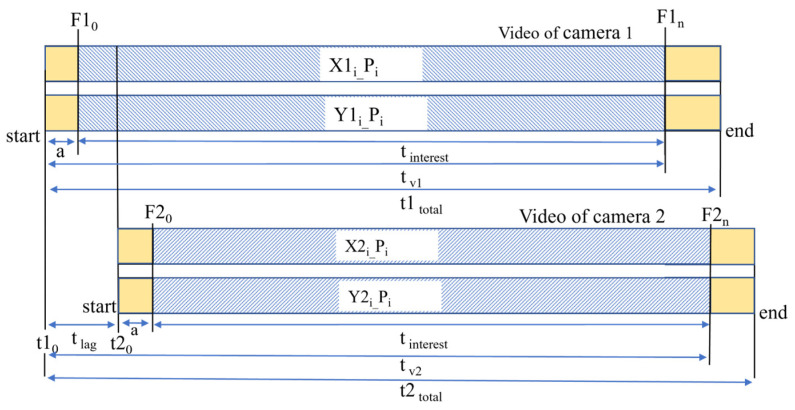
Visual illustration of the time lag between two videos.

The total video length recorded by camera-1 is denoted as (*t*1*_total_*), and (*t*2*_total_*) for the video recorded by camera-2. Because the cameras are operated manually, there will always be a time lag (*t_lag_*) between the cameras. According to Figure 7, camera-2 starts recording later than camera-1; hence, the video time of camera-2 (*t_v_*_2_) is the sum of the time lag (*t_lag_*) and the video time of camera-1(*t_v_*_1_) and can be expressed using Equation (11).

(11) $$t_{v2}=t_{v1}+t_{lag}$$

where $t_{v1}=a+t_{interest}$, $t_{v2}=a+t_{lag}+t_{interest}$, and $t_{lag}={t2}_{0}-{t1}_{0}$.

It is not always appropriate to use the very first frame as a starting point for synchronization analysis. The range of frames needed for analysis (*t_interest_*) can be defined as per the users’ criteria. (F1_0_ and F2_0_) are (a) frames away from the very first frame (*t*1_0_ and *t*2_0_) of camera-1 and camera-2, respectively. (F1_0_ and F2_0_) and (F1_n_ and F2_n_) are the first and last frames required for synchronization analysis in camera-1 and camera-2, respectively. In other words, it is the time range in frames (*t_interest_*) that is used for analysis, as shown in Figure 7.

In this study, all videos are 60 fps recordings. At higher frame rates such as 240 fps, synchronization accuracy is expected to improve due to finer temporal resolution. The ±1 frame window corresponds to smaller time intervals (16.7 milliseconds at 60 fps versus 4.2 milliseconds at 240 fps), reducing discretization errors. However, shorter exposure times in higher frame rates may introduce increased noise, potentially affecting tracking accuracy. The quadratic polynomial order is expected to remain optimal since the fundamental geometric relationship is unchanged.

#### 2.3.4. Stereo Triangulation

Stereo triangulation is a fundamental practice in computer vision, where the spatial coordinates of an object in a scene are determined in three dimensions. This method works with the corresponding projections of an object with images taken from different perspectives. The method employed in this instance is called binocular disparity, which computes depth information using the positional differences in corresponding picture points, allowing for a 3D estimate [51]. However, in practice, the perfect convergence of sight lines is often disrupted by measurement noise, calibration errors, and computational precision constraints. The triangulation error, or the smallest distance between skew lines from each camera view, is the result of such non-convergence. Such non-convergence causes a triangulation error, defined as the minimum distance between skew lines from each camera view. The resulting error compromises the accuracy of the reconstructed 3D coordinates and negatively impacts the precision of subsequent measurements. A two-stage triangulation approach is introduced to improve the reconstructed 3D points. Optimal triangulation is employed in the first stage to minimize the reprojection MSE using stored data from the synchronization stage. The second stage uses midpoint triangulation [50], which deduces 3D coordinates by locating the midpoint of the shortest line segment that connects two non-intersecting sight lines. The midpoint technique efficiently reduces the triangulation error because it minimizes the total of all summed squared distances to both sight lines. This makes it particularly useful in time-critical applications.

### 2.4. Software Development

**Figure 8:**
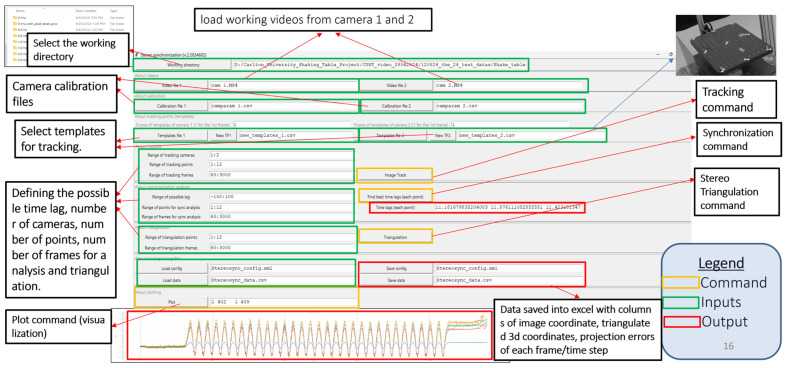
Graphical user interface of the developed vision-based 3D displacement measurement software.

**Figure 9:**
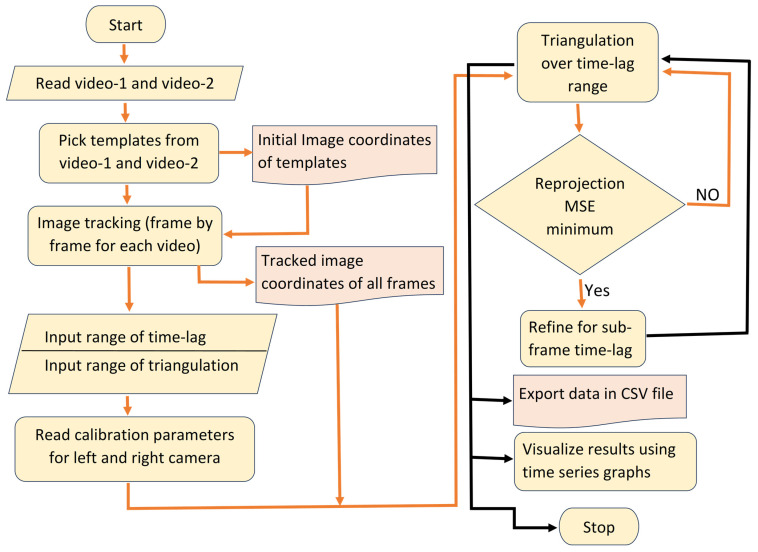
Flowchart of methodologies of the developed application software.

Developing a robust, well-organized software system is increasingly challenging for most civil engineers, who often lack formal software-engineering training and struggle with the complexities of modular design, version control, and testing procedures. Researchers can automate calibration, tracking, synchronization, and triangulation workflows, making measurement analysis far more efficient and accessible for structural engineers, especially when using consumer-grade cameras and IoT devices. We developed computer vision software in Python 3.12 for 3D displacement analysis. This software is specifically designed to analyze two videos of dynamic experiments that are not synchronized. The software captures the three-dimensional coordinates of multiple moving targets over a designated period. Tkinter is used to create a graphical user interface (GUI) for the program, as depicted in Figure 8 OpenCV is used as the main computer vision library, and NumPy and SciPy are used to execute mathematical operations. Users can access the folders containing their video files, calibration files, and templates. Users can define the range of tracking, synchronization, and triangulation points based on their needs. This setting allows users to select any frame required for tracking from the video file by simply inputting the number of frames. It allows users to save the analysis results in a CSV file, and they can visualize the time series displacements. The methodological flow chart of the 3D displacement measurement algorithm is shown in Figure 9. This software is freely available and can be downloaded from the GitHub repository at https://github.com/vin389/tkStereosync_v2 (accessed on 28 August 2025). The startup program is tkStereosync_v2.py.

Contact-based motion sensors, such as LVDT and accelerometers, provide motion data in only two directions, whereas vision sensors that employ stereo cameras provide spatial coordinates in three dimensions. Therefore, this software could be utilized as an additional measurement method in the field of earthquake engineering. Using this software, it is possible to analyze motion in six degrees of freedom, encompassing translation, rocking, rotation, and torsion. Furthermore, the deflection and strain modes of a particular set of structural components due to seismic loading can be computed by studying the distribution of three-dimensional positions of targets on the structure. This software can also be used in construction engineering testing facilities as a technique for non-destructive evaluation, allowing for the measurement of vibration, displacement, deflection, and even bending. In addition, it can be used to determine the deformation of the structure due to load or other environmental conditions, check the alignment of the structure, and track the growth of cracks to evaluate the structure’s condition and deterioration.

## 3. Results and Discussions

This section presents the experimental and theoretical results of the proposed vision-based displacement measurement and synchronization approach. The 3D positions of the points were measured for different scenarios and experimental setups.

### 3.1. Synchronization Method Performance Assessment Using Reprojection Errors

In the first experiment, the small-scale frame structure (mentioned in Section 2.1 as experimental program (a)) is used to assess the accuracy of the synchronization method by interpreting the triangulation projection error results. One quantitative metric for assessing the quality of three-dimensional reconstruction is the triangulation reprojection error. A lower reprojection error indicates better alignment between the computed disparity map and the actual scene geometry, which shows that the stereo image pair and the three-dimensional points are precisely aligned. This metric serves as an indicator of the accuracy with which the stereo vision system recovers the spatial position of the observed target. Ideally, two cameras are synchronized if the RMSE of triangulation is zero. However, zero error is ideal, not practical. Synchronization is critical in ensuring accurate measurements, particularly in dynamic systems where precise time alignment of data from multiple sources is required.

Table 1 presents a summary of the reprojected RMSE values computed using the conventional method (frame-level synchronization) and the proposed approach. The proposed method reduces the RMSE by 4.91% to 78.97%. For example, in Test 5, the method refined the baseline time lag from 99 to 99.28 frames, a 0.28 (4.67 milliseconds) frame correction. This refinement significantly reduced the baseline RMSE from 0.92 to 0.193 pixels, a 78.97% reduction. Across all tests, the method consistently improved triangulation accuracy, demonstrating that enhanced temporal synchronization significantly reduces triangulation reprojection error and improves stereo vision-based 3D displacement estimation quality. The effect of temporal refinement is more pronounced in dynamic high-frequency motion tests.

**Figure 10:**
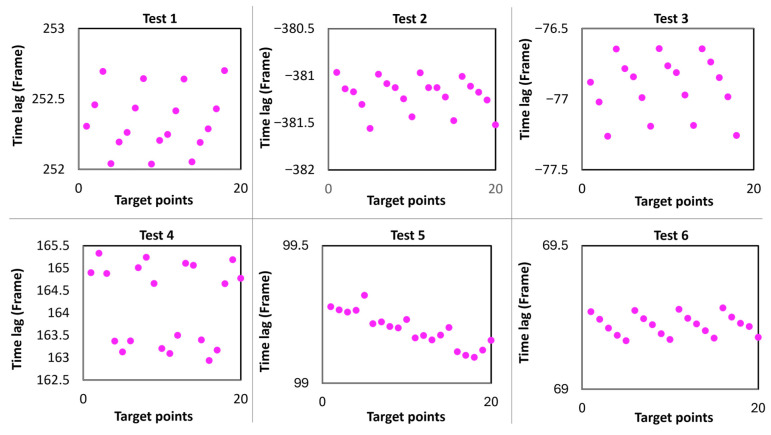
Point-specific time lag computation.

Conventional software-based synchronization methods apply a uniform time lag across all points within the triangulation process. However, in dynamic experimental scenarios, this approach results in significant reprojection errors due to the varying temporal characteristics of different scene elements. The proposed methodology addresses this limitation by implementing point-specific time lag correction, as shown in Figure 10, where each triangulated point is assigned an individual temporal offset based on its dynamic behavior. The results show that the reprojection error has a direct influence on the distribution of time lag across target points. When the reprojection error is large, the variation in time lag between points is also large, and vice versa. Test 4 in Figure 10 shows a 2.39-frame (39.833 milliseconds) time lag, resulting in a high reprojection RMSE of 0.99 pixels. Conversely, Test 5’s minimal 0.114-frame (1.902 milliseconds) lag yields a low RMSE of 0.114 pixels, demonstrating that minimizing reprojection errors enhances temporal alignment accuracy.

After synchronization, the time lag for all points should be identical. This ideal situation is only possible with global shutter cameras, as they eradicate rolling shutter issues by simultaneously capturing the entire frame. Unlike rolling shutter sensors, which read out the frame line by line, global shutters expose all pixels simultaneously, eliminating temporal skew. That being said, global shutter cameras are still comparatively costly and less available in the consumer market. Figure 10 illustrates the time lag for each point post-synchronization. This phenomenon exists due to the rolling shutter effect. A constant time lag across all points exacerbates rolling shutter error. Hence, to minimize this kind of error, the proposed algorithm computes a time lag for each point. The cameras used are of the rolling shutter type, which captures an image line by line, similar to scanners, which causes the image to distort if either the camera or the scene is in motion. Most rolling shutter cameras scan images from top to bottom with a fixed line delay [52,53]. Therefore, we partially solved measurement errors induced by the rolling shutter effect by assigning a point-specific time lag.

### 3.2. Method Validation Based on Small-Scale Structure Displacement

**Figure 11:**
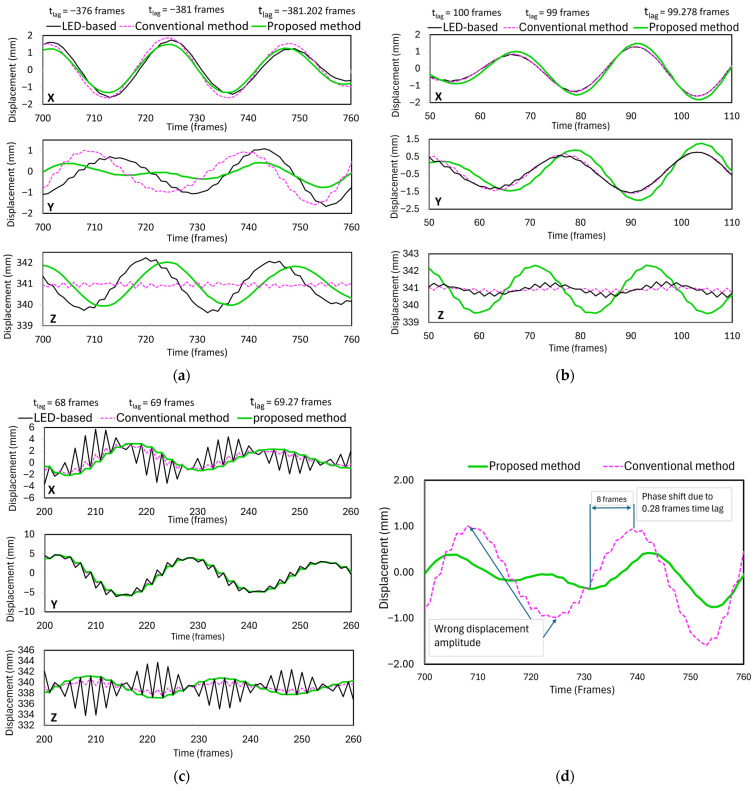
Focused time series 3D displacement results comparing LED-based, conventional, and proposed synchronization methods: (**a**) test 1, (**b**) test 5, and (**c**) test 6. (**d**) Zoomed illustration of the phase shift and erroneous displacement due to frame-level synchronization.

The 3D time series displacements of the small-scale structure mentioned in Section 2.1 were computed using three synchronization methods: light-emitting diode (LED)-based, conventional (frame-level), and proposed (sub-frame-level) synchronization methods. Then, the computed three-dimensional displacements were compared with each other. The LED-based synchronization method involves estimating the temporal offset between stereo video sequences using visual inspection of videos. The “Jump to Time” extension in the VLC media player was used for this purpose, enabling near-precise temporal alignment between video streams using an LED flashlight as a benchmark. The displacement trajectories shown in Figure 11a–c illustrate the results from the application of the three synchronization methods in tests 1, 5, and 6, characterized by the most significant reduction in reprojection RMSE. Consistent features are evident in both the synchronization phase alignment and measurement precision across all three spatial dimensions. From tests 1 and 5, amplitude and phase shift errors indicate that even sub-frame time delays can cause significant displacement errors (see Figure 11d). Time delays of 0.202 (3.370 milliseconds) and 0.278 frames (4.638 milliseconds) resulted in peak displacement differences of approximately 0.4 to 0.6 mm in the X and Y axes (in-plane). This effect was more substantial in the Z-axis (out-of-plane), where the deviations were almost 1 mm. Figure 11b illustrates this effect. These results demonstrate that even the most minor synchronization errors can lead to significant phase shifts (misplacing the displacement trajectory in time) and amplitude changes, as shown in Figure 11d, where a 0.28-frame time lag caused an 8-frame phase shift and up to a 1 mm displacement error, ultimately deteriorating displacement tracking. This is crucial in scenarios that require exact measurements of structures, such as those involving torsional effects, where the accuracy of measurement depends on the accuracy of the measurement of rotation angles and positions.

Figure 11c shows the displacement results of test 6. These results highlight the significant effect of time synchronization on measurement accuracy. The LED-based method introduces abrupt noise to the displacement signals with a 68-frame time lag. The deviations reached up to 6 mm in both the *X* (in-plane) and *Z* (out-of-plane) directions. The conventional method exhibited clear oscillations, with a time lag of 69 frames. However, the noise amplitude was slightly lower than that of the LED-based method. Although it appears to be more stable, it still does not provide a precise and reliable displacement signal due to residual time misalignment, as observed in previous studies using non-synchronized video sources [22]. However, the proposed method has a time lag of 69.27 frames and offers a smooth, continuous displacement profile. It closely follows the expected motion, with an obvious curvature and much less noise. These results demonstrate that even minor synchronization errors can lead to significant phase shifts and amplitude distortions, ultimately misleading the interpretation of the dynamic behavior of structures.

### 3.3. Method Validation Based on Bridge Displacement Measurement

A small-scale bridge experiment described as experimental program (c) in Section 2.1 was employed to further validate the proposed method. Mocap is used as a baseline measurement to compare the methods. Table 2 summarizes the comparison of errors between the two methods. In the X-direction (in-plane), the proposed method achieved error reductions ranging from 6.74% to 94.25% across different frequencies, with the most notable performance at 0.5 Hz, showing MAE, ME, and RMSE reductions of up to 90.60%, 94.25%, and 94.21%, respectively. The Y-direction (out-of-plane, primary excitation direction) showed consistent error reductions, with MAE reduction ranging from 12.00% to 83.47%, ME reduction up to 89.45%, and RMSE reduction reaching 87.90%. The Z-direction (in-plane) exhibited more modest reductions, with MAE reductions ranging from 0.89% to 39.64%, ME reductions up to 43.03%, and RMSE reductions reaching 38.90%. However, some conditions in the Z-direction exhibited a slight deterioration in the ME and RMSE metrics. In the Z-direction, the expected displacement is negligible, which can be compromised by noise.

### 3.4. Method Validation Based on Full-Scale Structure Displacement Measurement

**Figure 12:**
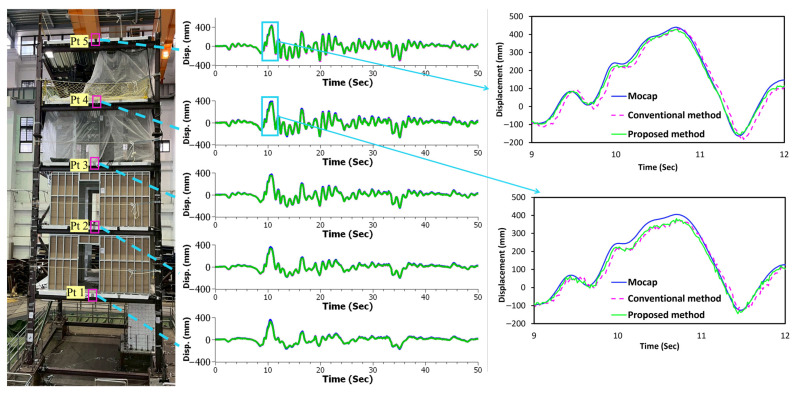
Schematic representation of a story-wise in-plane time series displacement.

Full-scale experimental validation represents a critical step in assessing the practical applicability and real-world performance of vision-based displacement measurement techniques as it introduces realistic challenges such as environmental conditions, structural complexity, and measurement distances that are absent in controlled laboratory settings. The results from the five-story structure experiment demonstrate substantial improvements in measurement accuracy across all three directional components (see Table 3) when evaluated using MAE and RMSE metrics. In the X-direction (in-plane), the proposed method achieved consistent MAE reductions ranging from 6.94% to 59.60% across all five measurement points, with corresponding RMSE reductions ranging from 1.08% to 48.02%. Point 5 (roof level, see Figure 12) exhibited the most significant enhancement, with the MAE reducing from 98.39 to 39.75 mm, representing a reduction of nearly 60 mm in absolute error. The Y-direction (out-of-plane) measurements, representing the most challenging measurement scenario, showed substantial MAE reductions ranging from 5.78% to 55.80%, with RMSE reductions reaching up to 58.75%. Point 5 again demonstrated exceptional performance, with the MAE decreasing from 656.41 to 290.13 mm, achieving a remarkable absolute error reduction of over 360 mm. In the Z-direction (vertical), the proposed method yielded MAE enhancements from 4.40% to 64.89% and RMSE reductions up to 62.86%, with Point 5 showing the most dramatic reduction, where MAE decreased from 410.05 mm to 143.98 mm, representing an absolute error reduction exceeding 260 mm. The measurement data reveal a clear trend in which the maximum deformation of the structure increases with height, and the MAE increases from the lowest point to the highest point. This demonstrates that errors are directly proportional to displacement magnitude, indicating that the MAE increases proportionally when the displacement is large. Although the absolute measurement errors remained substantial due to the challenging experimental conditions, including camera distance, lighting issues, the absence of distinguishable markers, and environmental noise, the proposed method consistently outperformed the conventional approach across all directional measurements, with the most pronounced improvements observed at the top of the structure, where substantial absolute error reductions were achieved in all three directions. Figure 12 illustrates how the proposed method (green line) improves displacement results by reducing temporal shifts and noise compared with the conventional method (pink line). This highlights the benefits of point-specific and sub-frame-level synchronization.

When monitoring large structures from a distance, consumer-grade 4K action cameras (3840 × 2160 pixels) with ultra-wide lenses (up to 150°) pose serious measurement challenges. Due to limited spatial sampling, each pixel represents a displacement of several millimeters, resulting in tiny tracking templates that are susceptible to noise, drift, and changes in appearance. These problems are made worse by their optical parameters, since ultra-wide lenses compromise spatial resolution for coverage, and lens distortion introduces systematic errors that are more noticeable at the edges of images. Wide-angle distortion, limited photon capacity, and sensor noise reduce measurement fidelity, even though sub-pixel techniques can refine estimates to millimeter levels. The attainment of high-accuracy displacement measurements is ultimately hampered by these intrinsic limitations, as well as calibration, tracking, synchronization, and triangulation.

### 3.5. Accuracy Assessment Based on Shaking Table Displacement Measurement

Additional verification was performed using the Mocap system as the primary reference, supplemented by a dial gauge for further verification. The evaluation was conducted under four shake table frequencies: 0.1, 0.5, 1.0, and 2.0 Hz. The frequency and amplitude of the shake table motion were kept constant throughout the video recording period to ensure consistent test conditions. The camera used in the experiment features an image sensor that measures 7.81 mm diagonally and provides a maximum effective resolution of 12 megapixels (4000 by 3000), with a square pixel size of 1.55 µm by 1.55 µm. The focal length of the camera is 3.3 mm. Although the experiments were conducted using specific consumer-grade cameras (SJCAM), the proposed methodology is not hardware-specific and can be applied to a broad range of camera models, provided they meet basic requirements such as adequate resolution, frame rate, and calibration capability. The results demonstrate a high level of consistency and agreement between the proposed method and the reference instruments, indicating its accuracy and applicability for structural displacement measurements under controlled dynamic conditions.

The proposed method exhibited minimal deviation from the Mocap results in the X-direction (in-plane), with maximum differences of 0.05 and 0.038 mm at 0.1 and 1.0 Hz, and 0.038 mm at 0.5 and 2.0 Hz, respectively. These discrepancies fall well within submillimeter precision thresholds, which are essential in structural dynamics experiments. In the Y-direction (out-of-plane) of the primary axis of shake table excitation, the proposed method is closely aligned with both the Mocap and dial gauge measurements. The maximum deviation recorded was 0.15 mm, observed when compared to the dial gauge at 0.1 Hz, while the differences relative to Mocap remained between 0.05 and 0.10 mm. These small variations reflect the accuracy of the proposed method and highlight the inherent resolution limitations of mechanical instruments, such as dial gauges. All methods reported low values in the Z-direction (vertical), where minimal displacement was expected; however, at higher frequencies, deviations were slightly more pronounced. The largest observed difference between the proposed method and Mocap was 0.097 mm at 1.0 and 2.0 Hz, indicating that small out-of-plane vibrations become more challenging to capture at increased motion speeds. Nonetheless, even in these cases, the proposed method maintained submillimeter accuracy. Overall, the proposed method consistently tracked displacement with high fidelity across all spatial directions and excitation frequencies. Its robust performance across a range of dynamic conditions, achieving a maximum discrepancy of just 0.20 mm (in comparison with the dial gauge in the Y direction), demonstrates its capability to deliver measurement precision comparable to that of laboratory-grade instruments. This level of accuracy is achieved using consumer-grade imaging equipment, highlighting the method’s potential for cost-effective, high-precision structural displacement monitoring in both laboratory and field environments.

**Figure 13:**
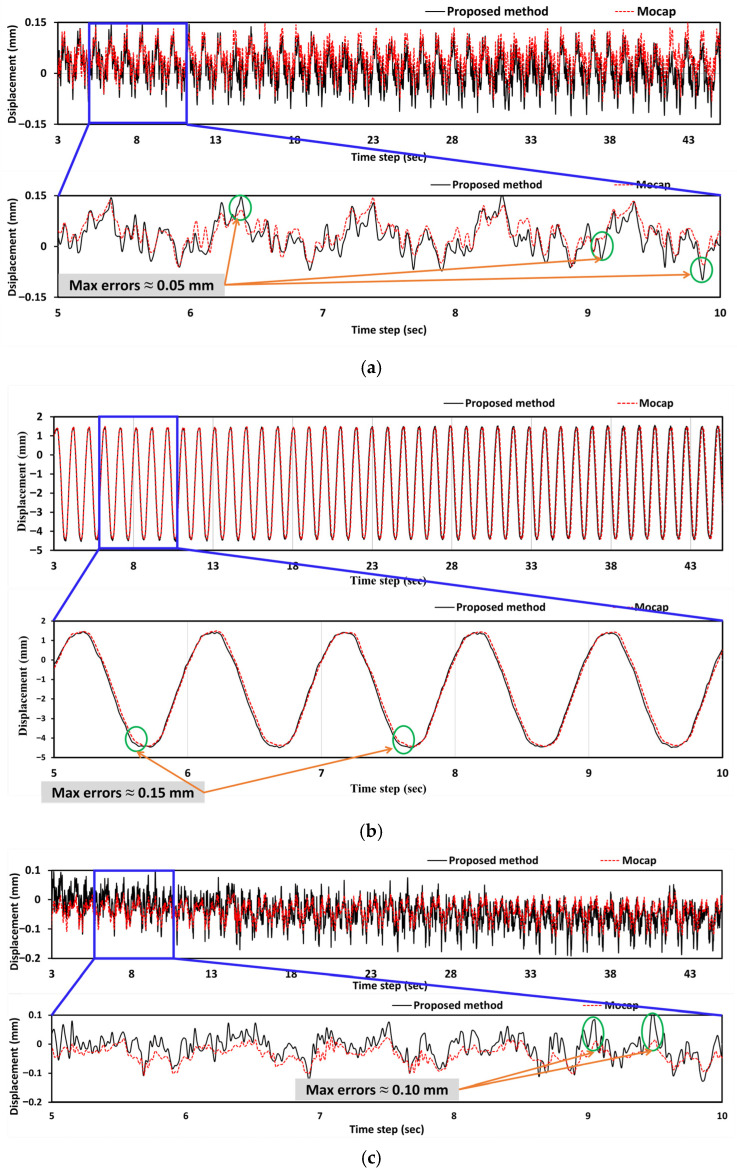
Displacement comparison between the proposed method and Mocap for a selected 1.0 Hz shake table frequency: (**a**) X-direction, (**b**) Y-direction, and (**c**) Z-direction.

Figure 13 presents the displacement time series data for a selected point under a shaking table excitation of 1.0 Hz. The X-direction displacement (Figure 13a) exhibits a high-frequency oscillatory behavior with relatively small amplitudes (±0.15 mm), characterized by complex vibrational patterns containing multiple frequency components. The proposed method achieves peak errors of 0.05 mm, demonstrating exceptional precision in capturing intricate lateral dynamics, including higher-order vibrational modes. The Y-direction (out-of-plane) response (Figure 13b) represents the primary motion axis, with large-amplitude sinusoidal displacement patterns (5 mm peak-to-peak) reflecting the principal excitation direction, where the proposed method maintains excellent tracking accuracy with peak errors limited to approximately 0.15 mm while preserving consistent phase alignment throughout multiple cycles despite the large displacement magnitudes, indicating that the method effectively addresses depth measurement issues that are typically challenging in traditional stereo vision methods. The Z-direction (vertical) measurements (Figure 13c) show intermediate-amplitude oscillations (±0.1 mm) with complex patterns. The peak errors reach approximately 0.10 mm. This three-dimensional analysis reveals that the proposed method successfully captures the complete dynamic response across all degrees of freedom with measurement errors remaining within acceptable limits for structural monitoring applications, effectively tracking the complex multi-directional interactions that reflect the three-dimensional nature of structural dynamics, where the primary excitation couples with secondary responses in orthogonal directions.

### 3.6. Effect of Vibration Frequency on Measurement Accuracy

**Figure 14:**
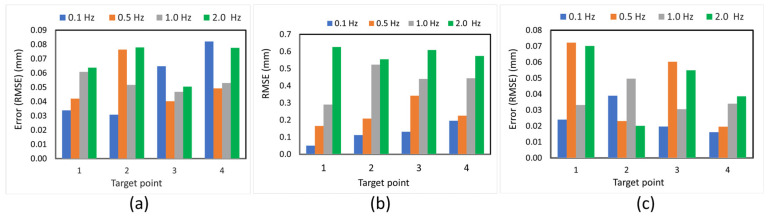
Comparison of displacement between the proposed method and Mocap for a selected 1.0 Hz shake table frequency: (**a**) X-direction, (**b**) Y-direction, and (**c**) Z-direction.

Figure 14 illustrates the impact of excitation frequency on measurement accuracy across all three spatial directions, utilizing Mocap as the ground-truth reference. The primary motion occurs in the Y-direction (out-of-plane), where the displacement is largest, and measurement errors exhibit strong frequency dependence. As the excitation frequency increases from 0.1 Hz to 2.0 Hz, the RMSE rises significantly, reaching a maximum of 0.626 mm at 2.0 Hz. This increase is attributed to the limitations of the stereo vision system’s temporal resolution, which is affected by the camera’s rolling shutter and becomes less effective at tracking fast displacement changes. In contrast, the X- and Z-directions (both in-plane) exhibit only minor vibrations due to the unidirectional motion of the shake table. Nonetheless, errors in these directions remain below 0.082 mm and 0.072 mm, respectively, indicating that the proposed synchronization method maintains submillimeter accuracy even for secondary motion components. Overall, the results demonstrate that while measurement accuracy decreases with increasing excitation frequency, especially in the primary motion axis (Y), the proposed system still achieves high precision across all tested frequencies.

### 3.7. Structural Higher-Mode Vibration Observation

**Figure 15:**
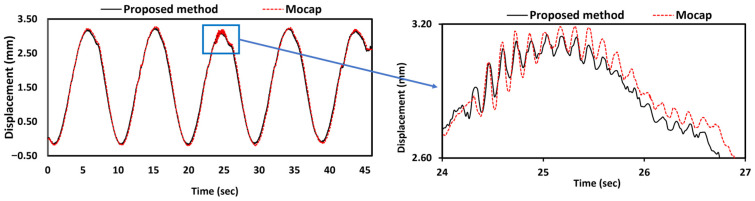
Measurement accuracy for higher vibration frequency.

Figure 15 shows that the proposed method accurately captures high-frequency structural vibrations with submillimeter displacements, revealing a complex, multiscale dynamic response with two distinct frequency components. The displacement time series exhibits large-amplitude oscillations (2.85 mm peak-to-peak) at the fundamental excitation frequency of 0.1 Hz, representing the forced response of the bridge structure to the shake table input. Superimposed on this response are high-frequency internal vibration components with an amplitude of approximately 0.1 mm occurring at an estimated frequency of 8.0 Hz. The magnified view demonstrates exceptional measurement accuracy, with the proposed method achieving an average difference of 0.0471 mm and a maximum difference of 0.108 mm when capturing these rapid displacement variations compared to the Mocap system. Although slight discrepancies are observed in the zoomed region, they are likely attributed to the behavior of the ECC tracking algorithm under rapid structural motion rather than measurement noise. Through iterative refinement within a parametric warp space, ECC is optimized for smooth, incremental image alignment. Although it performs well under a broad range of conditions, its responsiveness to sharp, high-frequency motion may be limited by the convergence dynamics of the optimization process [4]. Nonetheless, the successful resolution of 8.0 Hz vibrations demonstrates that the proposed synchronization method effectively overcomes the limitations of stereo vision in terms of temporal resolution. This enables accurate measurement across a wide frequency range (0.1–8.0 Hz) via point-specific time lag correction and shows excellent agreement with the Mocap system. The consistency observed throughout both low-frequency primary motion and high-frequency secondary vibrations confirms the reliability and practical applicability of the synchronization approach for real-world structural monitoring scenarios where multiple vibration modes may be simultaneously excited.

**Figure 16:**
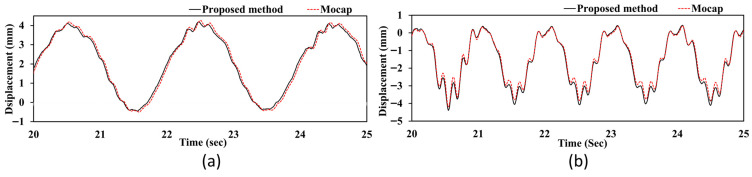
Bridge vibration response comparison between proposed method and Mocap: (**a**) 0.5 Hz; (**b**) 1.0 Hz.

The proposed method offers a reliable approach for observing and analyzing how a system responds structurally under various excitation conditions. Figure 16 shows the time history of the structural vibration of the bridge system. This data was analyzed using the proposed method and compared with the motion capture system. The results indicate that the structural frequency of the bridge increases as the excitation frequency increases. This demonstrates that the proposed method can effectively track dynamic changes in structural behavior under various excitation conditions. Figure 16a shows that the structural vibration has a lower frequency when the shake table runs at 0.5 Hz. Figure 16b shows a higher structural vibration frequency when the shake table frequency is increased to 1 Hz. The strong correlation between the proposed method and the reference motion capture system at both frequency levels confirms the reliability of the stereo vision approach in capturing frequency-dependent structural responses. This ability is crucial for structural health monitoring, in which the dynamic characteristics of the system can change under different loading conditions. The proposed method successfully maintains measurement accuracy while monitoring these variations in real time.

## 4. Limitations

The quality of point tracking is fundamentally the constraint on the precision of our synchronization method. Motion blur degrades the high-frequency details used by ECC correlation, leading to faulty displacements and reduced MSE curves. Partial occlusion generates spurious mismatches that distort alignment and triangulation error profiles. Both phenomena cause polynomial interpolation to produce faulty time lag estimates. While the ECC tracker neither failed nor required human intervention, marker size, video resolution, standoff distance, field of view, baseline distance, and environmental noise still affected tracking accuracy. These factors inherently impose their own limitations on displacement accuracy, which directly translates into synchronization accuracy.

The GUI software is limited to two cameras and requires the user to define their time lag search ranges; poorly chosen ranges can cause incorrect synchronization, consequently compromising the displacement results. This method assumes consistent inter-camera frame rates and relies on standard video codecs, limiting compatibility with proprietary recording formats. The tracking system processes sequentially, resulting in high computational complexity with an approximate processing time of 1–2 min per 1000 frames, depending on the video resolution. Furthermore, the software does not support real-time processing. The calibration framework necessitates predefined parameter structures without cross-platform compatibility or automated quality assessment, potentially compromising triangulation accuracy when suboptimal calibration data is employed. Further details are included in the software’s documentation.

## 5. Conclusions

This study presents a novel point-specific synchronization method for stereo vision systems that significantly enhances the accuracy of three-dimensional (3D) displacement measurements in dynamic structural monitoring applications. The proposed approach addresses the fundamental limitations of conventional frame-level synchronization by implementing individual temporal offset corrections for each reconstructed point through a two-phase optimization process using polynomial interpolation techniques that combine coarse frame-level synchronization with sub-frame precision refinement. This methodology accounts for the varying temporal characteristics of different scene elements under dynamic loading conditions while maintaining the imaging process’s inherent geometric relationships. Furthermore, the proposed method mitigates the rolling shutter effect and ensures precise temporal synchronization for accurate motion analysis.

The comprehensive experimental validation across four distinct test configurations, namely small-scale frame structure, shake table system, bridge model, and full-scale experimental structure, demonstrates substantial improvements in measurement accuracy. The rigorous evaluation employed various excitation frequencies (0.1–2.0 Hz) and diverse tracking templates to assess method robustness under varying conditions. The proposed method achieved RMSE reductions ranging from 4.91% to 78.97% compared with conventional synchronization approaches. Comprehensive validation against Mocap systems and dial gauge measurements confirms the method’s practical accuracy, with displacement RMSE reductions of up to 0.69 and 240 mm for small- and full-scale experiments, respectively. Additionally, the proposed method successfully captures complex multiscale dynamic responses, including high-order structural vibrations up to 8.0 Hz with submillimeter precision.

In addition, we successfully developed user-friendly GUI software for determining 3D displacement using stereo vision technology. It is challenging to build a fully functional software system that combines various libraries for calibration, target selection, tracking, synchronization, triangulation, and visualization, especially for civil engineers and researchers who may lack strong programming skills. This tool fills that gap by offering an easy-to-use interface. This enables researchers to use advanced computer vision techniques for measuring 3D displacement without the need for in-depth programming knowledge. This makes advanced displacement analysis easier for the engineering community.

The practical implications of this research extend beyond laboratory validation to real-world structural health monitoring applications where precise displacement tracking is critical for safety and performance evaluation. Despite the poor measurement accuracy in the full-scale experiment, the proposed method significantly reduces displacement errors. Its successful validation across a wide frequency range (0.1–8.0 Hz) using consumer-grade imaging equipment demonstrates the method’s cost-effectiveness while maintaining measurement precision comparable to laboratory-grade instruments. The method’s ability to suppress measurement noise while preserving temporal fidelity, combined with accurate capture of both primary motion and high-frequency secondary vibrations, establishes its suitability for critical applications such as bridge monitoring, seismic response analysis, and vibration-based damage detection systems.

Although the current study demonstrates robust performance across diverse experimental configurations with comprehensive validation against industrial-grade reference systems (Mocap), future research should investigate the method’s performance under varying environmental conditions, including different lighting scenarios and weather conditions. The observed frequency-dependent error characteristics, particularly the influence of rolling shutter effects at higher frequencies, warrant further investigation to establish optimal camera specifications and frame rates for specific monitoring applications. The development of automated target detection and tracking algorithms that can handle various template types (high-contrast markers, natural features, and stickers) could further enhance the practical applicability of the proposed synchronization approach for large-scale infrastructure monitoring. The integration of machine learning techniques for dynamic time lag prediction based on scene characteristics, camera configuration, and motion patterns represents a promising avenue for advancing real-time adaptive synchronization in stereo vision-based displacement measurement systems.

This study’s findings contribute to the growing body of knowledge in computer vision-based structural monitoring by providing a robust solution for temporal synchronization challenges. The proposed methodology offers a significant step forward in achieving the measurement accuracy required for critical infrastructure monitoring applications, where precise displacement quantification is essential for informed decision-making regarding structural integrity and safety.

## Figures and Tables

**Table 1:** Comparison of triangulation RMSE between proposed and conventional methods.

Case	Conventional Method (CM)			Proposed Method (PM)			RMSE Reduced Relative to CM (%)
	Time Lag (Frame)	RMSE (Pixel)		Time Lag (Frame)	RMSE (Pixel)		
		Camera-1	Camera-2		Camera-1	Camera-2	
1	−381	1.427	0.980	−381.20	0.571	0.391	59.99
2	252	0.324	0.378	252.35	0.295	0.344	8.98
3	−77	0.269	0.299	−76.88	0.256	0.284	4.91
4	165	1.154	1.125	164.90	0.988	0.992	14.37
5	99	0.920	0.943	99.28	0.193	0.198	78.97
6	69	0.152	0.167	69.27	0.109	0.120	38.7

Note: The “conventional method” refers to the frame-level synchronization throughout the paper.

**Table 2:** Statistical summary of errors and comparison between the conventional and proposed methods of the small-scale bridge experiment. (NB: units are in millimeters).

X-Direction	Conventional Method			Proposed Method			Error Reduced (%)		
Target	MAE	ME	RMSE	MAE	ME	RMSE	MAE	ME	RMSE
0.1 Hz	0.11	0.03	0.13	0.08	0.02	0.10	21.00	28.30	26.65
0.5 Hz	1.17	0.63	0.73	0.11	0.04	0.04	90.60	94.25	94.21
1.0 Hz	0.16	0.04	0.05	0.15	0.04	0.05	6.74	−0.65	2.81
2.0 Hz	1.29	0.10	0.18	0.22	0.07	0.09	83.20	30.41	53.42
Y-direction									
0.1 Hz	0.47	0.2	0.23	0.14	0.04	0.05	71.12	79.55	77.92
0.5 Hz	1.54	0.74	0.84	0.36	0.08	0.1	76.9	89.45	87.9
1.0 Hz	0.78	0.15	0.19	0.69	0.15	0.19	12	−0.32	−0.28
2.0 Hz	2.66	0.19	0.44	0.44	0.1	0.14	83.47	48.18	69.08
Z-direction									
0.1 Hz	0.10	0.04	0.05	0.10	0.04	0.05	2.86	−5.53	−3.33
0.5 Hz	0.26	0.10	0.12	0.17	0.06	0.07	35.04	43.03	38.90
1.0 Hz	0.16	0.07	0.07	0.16	0.07	0.08	0.89	−8.85	−7.96
2.0 Hz	0.22	0.06	0.07	0.13	0.06	0.07	39.64	1.24	1.72

**Table 3:** Statistical summary of errors and comparison between the conventional and proposed methods of the full-scale experiment. (NB: units are in millimeters).

X-Direction	Conventional Method			Proposed Method			Error Reduced (%)		
Target	MAE	ME	RMSE	MAE	ME	RMSE	MAE	ME	RMSE
Point 1	42.99	8.66	8.14	40.01	7.66	8.05	6.94	11.54	1.08
Point 2	46.85	8.84	10.21	28.87	6.74	7.89	38.38	23.69	22.74
Point 3	52.09	8.67	10.49	27.67	8.10	9.32	46.88	6.64	11.15
Point 4	79.34	13.92	16.81	54.94	13.44	16.59	30.75	3.45	1.30
Point 5	98.39	14.85	18.98	39.75	8.02	9.87	59.60	46.01	48.02
Y-direction									
Point 1	205.35	24.42	102.80	166.36	20.85	85.08	18.98	14.61	17.24
Point 2	215.23	46.37	54.80	147.76	35.04	40.58	31.35	24.42	25.95
Point 3	260.39	38.88	52.34	165.53	45.64	51.09	36.43	−17.39	2.39
Point 4	244.88	29.92	57.27	230.74	36.11	49.79	5.78	−20.69	13.07
Point 5	656.41	93.17	121.02	290.13	40.38	49.92	55.80	56.66	58.75
Z-direction									
Point 1	30.36	4.37	5.42	27.17	4.63	5.50	10.52	−5.93	−1.62
Point 2	14.08	2.43	3.15	13.46	1.80	2.54	4.40	25.73	19.43
Point 3	60.76	12.17	14.67	35.33	11.32	12.70	41.86	7.00	13.46
Point 4	102.04	13.36	24.95	70.03	16.17	20.97	31.36	−21.03	15.95
Point 5	410.05	41.36	78.02	143.98	19.27	28.98	64.89	53.40	62.86

## Data Availability

The raw data supporting the conclusions of this article will be made available by the authors upon request.
